# A pilot study on the prevalence of lice in Irish beef cattle and the first Irish report of deltamethrin tolerance in Bovicola bovis

**DOI:** 10.1186/s13620-021-00198-y

**Published:** 2021-07-05

**Authors:** Fiona Mckiernan, Jack O’Connor, William Minchin, Edward O’Riordan, Alan Dillon, Martina Harrington, Annetta Zintl

**Affiliations:** 1 School of Veterinary Medicine, University College Dublin, Belfield, Dublin 4, Ireland; 2 MSD Animal Health, Dublin, Ireland; 3 Teagasc, Animal & Grassland Research and Innovation Centre, Grange, Dunsany, Co. Meath Ireland; 4 Teagasc Moorepark, Fermoy, Co. Cork Ireland; 5 Teagasc Oakpark, Co. Carlow, Ireland

**Keywords:** Lice, Pediculosis, Ectoparasiticide, Deltamethrin, Resistance

## Abstract

**Background:**

Pediculosis in cattle causes significant itching, irritation and stress to the animal, often resulting in skin damage and poor coat condition. The control of bovine pediculosis in Ireland is based predominantly on commercial insecticides belonging to one of two chemical classes, the synthetic pyrethroids and the macrocyclic lactones. In recent years, pyrethroid tolerance has been reported in a number of species of livestock lice in the United Kingdom and Australia.

**Results:**

In this pilot survey, lice were detected in 16 (94%) out of 17 herds visited. Two species of lice, *Bovicola bovis *and *Linognathus vituli *were identified*. In vitro *contact bioassays showed evidence of deltamethrin tolerance in *Bovicola bovis* collected from 4 farms. This was confirmed by repeatedly assessing louse infestations on treated animals on one farm.

**Conclusions:**

To our knowledge this is the first record of insecticide tolerant populations of lice in Irish cattle. The results also provide new data on the species of lice infesting beef cattle in Ireland and the prevalence and control of louse infestations in Irish beef cattle herds.

## Introduction

**Fig. 1:**
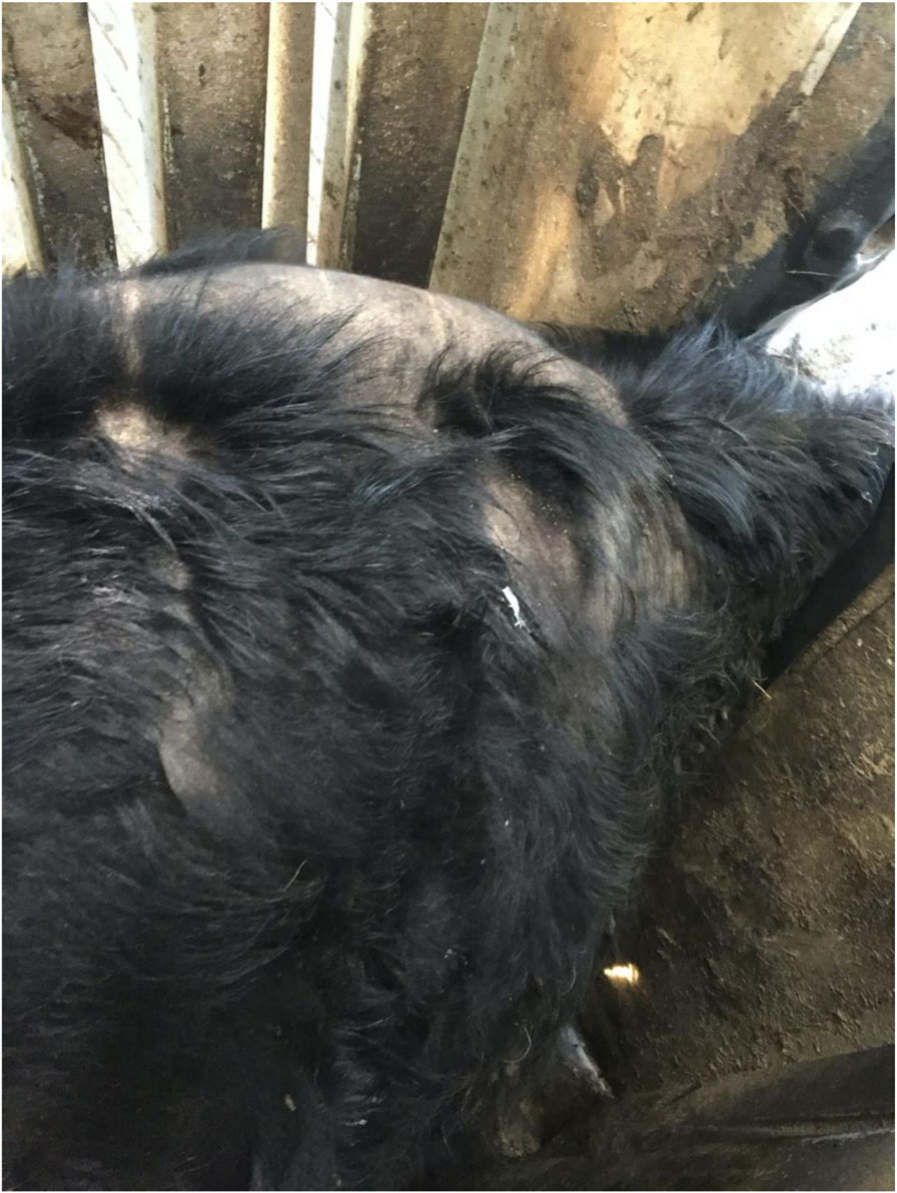
A dorsal view of the shoulders of a heifer showing the coat damage caused by a heavy infestation of *Bovicola bovis* lice

Infestations of lice, also known as pediculosis, are more common in cattle than any other domestic animal [1]. Whilst light infestations of both sucking and biting lice often go unnoticed and are not usually considered to be of clinical importance, heavier infestations cause pruritis, which in turn leads to hair loss and skin damage often resulting in poor quality leather hides and significant economic losses to the producer [2]. Moreover, the restlessness and stress associated with pediculosis can result in decreased appetite, decreased weight gain [3, 4] and decreased milk yields [3, 5] and should be considered an animal welfare issue. Heavy infestations with sucking lice can cause anemia [6, 7] which in some cases may be fatal [8, 9]. Figure 1 shows the coat damage of an animal sampled in our study who was heavily infested with *Bovicola bovis* lice.

Reports on the herd prevalence of louse infestations and the cattle louse species responsible have been published from various European countries including Sweden, Iceland, Norway, England and Scotland [10–14]. The only report on the prevalence of cattle lice in Ireland dates back to 1977 when Oormazdi and Baker investigated a single herd of adult cattle in a Dublin-based abattoir [15]. They recorded an infection rate of 56 % and the presence of 4 different louse species.

Control of bovine pediculosis in Ireland, as in Europe, relies primarily on the use of commercial ectoparasiticides belonging to either the synthetic pyrethroid or macrocyclic lactone chemical classes. In recent years, concerns have been raised globally over the development of insecticide resistance in louse populations of livestock associated with the widespread use of ectoparasiticides in agricultural production. To date, resistance has been reported in a number of species of lice including *Bovicola ovis, Bovicola ocellatus, Haematopinus tuberculatus* and *Haematopinus suis* [16–19]. In 2015, Sands and colleagues published the first reports of deltamethrin tolerance in the cattle louse *Bovicola bovis* in the UK [20].

The aims of this pilot study were to evaluate the prevalence of louse infestations and species in beef cattle and to assess the efficacy of the synthetic pyrethroid deltamethrin on lice collected from heavily infested herds.

## Materials and methods

### Farm visits, louse collection and identification

17 beef farms were recruited opportunistically for this study with the help of Teagasc (the Irish Agriculture and Food Development Authority), MSD Animal Health and staff at the UCD School of Veterinary Medicine. None of the herds were closed. Prior to sampling it was not known if the participating farms were likely to have lice or not nor whether the participating farms had had previous problems with the efficacy of any commercial louse management products. Farms were visited between January 2019 to March 2019 and November 2019 to March 2020. At the time of the visit, cattle had been housed for between 1 and 12 weeks. The treatment history of each animal since housing of that year was recorded. Each herd was sampled once, with the exception of farm 1, which was sampled on multiple occasions as described below. The number of animals selected for sampling on each farm ranged from 20 to 65 animals (5-100 % of the herd). The great variation was due to the opportunistic nature of the sampling, as visits generally coincided with a date when the farmer was putting animals through the crush for other reasons such as anthelmintic treatment. Samples were collected from 5 documented louse predilection sites: the withers, shoulder, topline, flank and the rump/tail area [21]. At each site an area twice the width of the comb (amounting to approximately 13cm^2^) was combed 15 times using a fine-tooth plastic headlouse comb. The combings including lice, bovine skin scurf, hair and other debris were collected in a 100mm diameter polystyrene petri dish. A new petri dish and clean plastic comb were used for each animal. Samples were transported to the laboratory and maintained in an unilluminated incubator at 30 °C and 75 % relative humidity (RH).

Lice were examined under a dissection microscope and identified to species, sex and lifecycle stage using the taxonomic keys provided by Lapage [21]. Adult female *Bovicola bovis* lice collected from 4 farms were retained for *in vitro* bioassays.

### Repeat farm visits to assess insecticide resistance in vivo

In order to assess the efficacy of ectoparasiticide treatment *in vivo* and to validate the results of the *in vitro* bioassays, 65 animals in farm 1 were sampled on four occasions over a space of 7 weeks between January 2019 and February 2019 coinciding with louse treatment dates using a louse control product of the farm manager’s choice, a deltamethrin-based pour-on. Animals were treated according to the manufacturers’ instruction. The same animals were sampled on each occasion. Samples were taken from each animal according to the sampling protocol described above and recorded as the number of days post-treatment for lice, beginning on ‘day 0’. Combings were analyzed as described above.

### In vitro contact bioassay to detect insecticide resistance in the cattle chewing louse Bovicola bovis

*In vitro* contact bioassays to assess the susceptibility of adult female *Bovicola bovis* to the synthetic pyrethroid deltamethrin were carried out as described by Levot & Hughes [22] with some modifications. Briefly, a stock solution of 50 mg/ml deltamethrin was prepared by dissolving 250 mg of 99.9 % deltamethrin (Pestanal® analytical standard, product no. 45,423, Sigma-Aldrich®, USA) in 5ml of 99 % acetone. Stock solutions were stored at -20 °C for up to three months. For the bioassay, dilutions of 10 mg/ml and 5 mg/ml deltamethrin were prepared from the stock solution in 99 % acetone. 99 % acetone was used as a negative control and 2.5 % tea tree oil (Optima**®** Australian tea tree) diluted in 99 % acetone was used as a positive control [20]. 0.5ml of each dilution was pipetted onto 55mm diameter Whatman filter paper disks, and the disks placed in 55mm diameter glass petri dishes in a fume hood for 30 min to allow the acetone to evaporate.

Once the filter papers had dried, 10 adult female *Bovicola bovis* lice were placed onto each of the disks using forceps. The lids were replaced and the petri dishes returned to the incubator. Observations and records of louse mortality were made at 30 min, 1, 2, 3, 4 and 24 h from the start of the incubation period. Lice were considered dead when there was no movement of the legs, mouthpiece, antennae or abdomen even when probed with a forceps. Bioassays for each deltamethrin concentration were performed in triplicate.

As a control, the bioassay was also performed on *Bovicola equi* lice collected from a horse. As deltamethrin-based ectoparasiticides are not licenced for commercial use in horses in Ireland, it was assumed that these lice would be less exposed and therefore highly susceptible to exposure to deltamethrin.

### Structured interviews with participating farmers

Prior to sampling, a structured interview was carried out with each farmer. In the interview, farmers were asked about the incidence of louse infestations in their herd, their use of commercial louse control products and whether they thought they were effective.

### Data analysis

The results of the *in vitro* bioassays were expressed as percentage mean louse mortality at the various insecticide concentrations (and controls ) ± standard error. For each bioassay, the number of lice surviving at 1 and 0.5 % deltamethrin were compared to those in acetone control at the 24-hour timepoint using one-way ANOVA. Dunnett’s multiple comparisons test was used for post-hoc analysis. In order to facilitate a more general comparison, the 24-hour mean louse mortalities were corrected using Abbott’s formula [23].

## Results

### Prevalence of louse infestation

Of the 17 farms that were visited, lice were detected in 16 (94 %), with 51 % (335) of 652 animals being positive for lice. The percentage of sampled animals that were positive for lice on each farm ranged from 10 to 100 %. 2 species of lice were identified, the chewing louse *Bovicola bovis* and the sucking louse *Linognathus vituli.* 88 % of infected animals were infested only with *B. bovis* and 5 % only with *L. vituli*. Mixed infestations of both *B. bovis* and *L. vituli* were found in 7 % of animals.

### Repeat farm visits to assess insecticide resistance in vivo

**Fig. 2:**
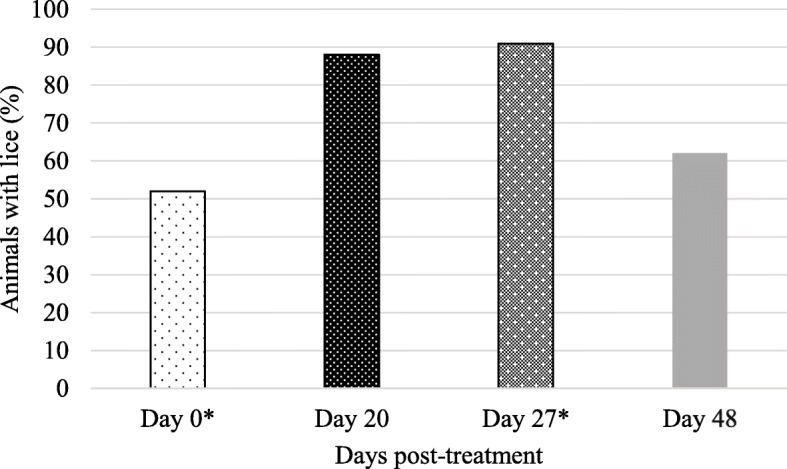
The prevalence of lice pre- and post-treatment with a deltamethrin-based pour-on (n = 65) (*indicates treatment dates)

On the farm where animals were sampled on four occasions to assess the efficacy of ectoparasiticide treatment *in vivo*, lice were present on 52 % of animals in the herd on day 0, prior to any louse treatments being administered. On day 20 post-treatment with a deltamethrin-based pour-on, 88 % of animals were positive for lice and on day 27 post-treatment, 91 % of animals were positive (Fig. 2). On this date (day 27), the farm manager treated all animals a second time using the same deltamethrin-based product as used previously on day 0. On day 21 (21 days after the second louse treatment), lice were present on 62 % of animals.

### In vitro contact bioassay

**Fig. 3:**
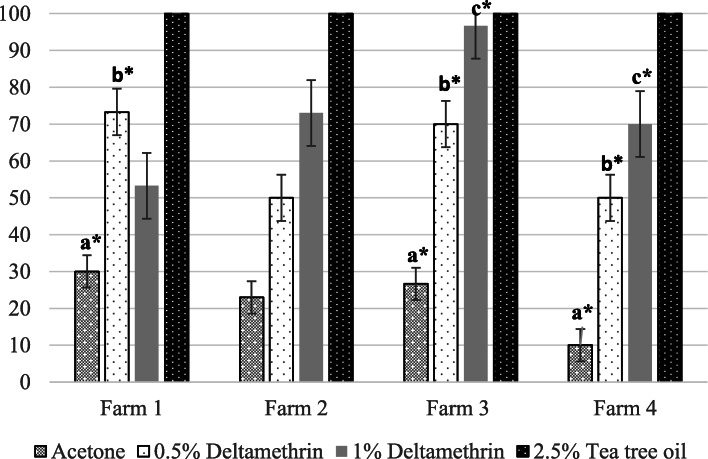
The 24-hour percentage mean louse mortality of *Bovicola bovis* (± SE) placed in contact with 1 % deltamethrin, 0.5 % deltamethrin, 99 % acetone control and 2.5 % tea tree oil control (different small letters indicate significantly different groups within each farm)

*In vitro* contact bioassays to assess susceptibility to deltamethrin were performed on *Bovicola bovis* lice collected from 4 farms. These 4 farms were selected for deltamethrin susceptibility testing as large numbers of *B. bovis* lice (> 100 lice per farm) were collected during the on-site sampling visits. The 24-hour percentage mean louse mortalities (without Abbott’s correction) for farms 1,2,3 and 4 are shown in Fig. 3.

On farm 1, there was a significant difference in the 24-hour louse mortalities between the experimental groups (1 % deltamethrin, 0.5 % deltamethrin, acetone & tea tree oil controls) (F = 13.84, *P* = 0.0016, df = 3). Analysis of Dunnett’s multiple comparisons post-hoc test showed that there was no statistical difference between the 24-hour mean louse mortalities in the 1 % deltamethrin assay and the acetone control (*P* = 0.1667) however there was a significant difference between the 24-hour mean louse mortalities of the 0.5 % deltamethrin and the acetone control assays (*P* = 0.0126).

On farm 2, there was a significant difference between mean louse mortalities in all of the groups (F = 5.207, *P* = 0.0276, df = 3) however post-hoc analysis showed that there was no significant difference in the mortalities of both, the 1 and 0.5 % deltamethrin groups and the acetone control at 24 h (*P* = 0.0924; *P* = 0.4564).

On farm 3 there was also a significant difference in the mean mortalities between all experimental groups (F = 24.31, *P* = 0.0002, df = 3). The mean louse mortalities at both the 1 % deltamethrin (*P* = 0.0003) and 0.5 % deltamethrin (*P* = 0.0054) assays were statistically different from that of the acetone control.

Similar results to farm 3 were observed on farm 4. There was a significant difference in the mortalities between all of the experimental groups (F = 19.00, *P* = 0.0005, df = 3) and post-hoc analysis showed that the 24-hour mean louse mortalities of the 1 % (*P* = 0.0031) and 0.5 % (*P* = 0.0284) deltamethrin assays were significantly higher than the acetone control.

Exposure of *Bovicola equi* lice to 1 and 0.5 % deltamethrin for 24 h resulted in 100 % mortality at both concentrations of deltamethrin.

In order to facilitate a more general comparison with published figures, louse mortality rates after 24 h of continuous contact with filter paper impregnated with deltamethrin were corrected using Abbott’s formula. At 1 % deltamethrin, the percentage mean louse mortality rates following Abbott’s correction were calculated as 33.34 % (± 8.6 %), 65.22 % (± 8.7 %), 95.45 % (± 3.8 %) and 66.67 % (± 8.6 %) in farms 1, 2, 3 and 4 respectively. At 0.5 % deltamethrin the corrected values were 61.9 % (± 8.9 %), 34.78 % (± 8.7 %), 59.09 % (± 9 %) and 44.4 % (± 9.1 %) for farms 1, 2, 3 and 4 respectively.

### Structured interviews with participating farmers

100 % of the participating farmers stated that they treat their cattle for lice each year at least once during the winter housing period. 14 (82 %) of the participating farmers had treated their cattle for lice in the 8 weeks prior to the sampling visit.

Two farms had used two louse management products containing different active ingredients on their cattle while the remaining 12 farms had used just one louse management product. Ivermectin-, cypermethrin- and deltamethrin-based products were most commonly used with ivermectin- and cypermethrin-based products being equal in popularity across the 14 farms (31.25 %) followed by deltamethrin-based products (25 %). Doramectin-based products were used on just 1 farm (6.3 %). One farmer used a diazinon-based sheep dip product as he found products licensed for use in cattle to be ineffective.

13 (76 %) farmers reported no problems with louse infestations within their herd or with the efficacy of commercial louse management products. However, 4 farmers stated they had experienced efficacy issues with various deltamethrin-based products requiring several treatments to be effective.

## Discussion

In this pilot study, lice were recorded on 94 % of farms and on 51 % of animals confirming anecdotal evidence that seasonal infestations of cattle herds with lice are very common in Ireland. This herd prevalence was similar to what has been reported in the UK (75–80 %) [13, 14], Sweden (93 %) [10] and Iceland (70 %) [11]. Two species of lice were identified in the present study, the chewing louse *Bovicola bovis* and the sucking louse *Linognathus vituli.* Similar to reports from the UK [13, 14, 24], *B. bovis* was by far, the more prevalent. In contrast, a previous study conducted by Oormazdi and Baker, identified four species of lice in a group of cattle in a Dublin-based abattoir; the chewing louse *Bovicola bovis* and the sucking lice *Solenopotes capillatus, Haematopinus eurysternus* and *Linognathus vituli* [15]. Previous studies in the UK and Iceland also reported *S. capillatus* from cattle herds [11, 14]. According to Craufurd-Benson, the prevalence of *S. capillatus* in cattle herds is commonly underreported due its small size [25]. Furthermore, the face and head of the animal, which has been identified as a common predilection site of *S. capillatus* [24, 25], was not examined in this pilot study for logistical and animal welfare reasons. Further work will be required to determine whether this louse species was overlooked in our study or whether it has become less prevalent.

Results of the structured interviews showed that farmers rely on a range of commercial ectoparasiticides including both synthetic pyrethroid, particularly deltamethrin-, and macrocyclic lactone-based products. However, a number of farmers indicated efficacy problems with the former. These observations were born out by the *in vitro* contact bioassays using deltamethrin which showed deltamethrin-tolerance in *B. bovis* lice collected from four farms.

It is important to point out that published information on the minimum concentration of synthetic pyrethroids required to kill lice in livestock is somewhat conflicting. With regard to *in vitro* contact bioassays to assess insecticide tolerance in lice, FAO guidelines state that ‘the survival of one or more louse at 5 mg/l (0.0005 %) or greater is taken as an indication of resistance’[26]. In contrast Boray and colleagues reported the minimum synthetic pyrethroid concentration required to kill *B. ovis* lice to be 0.5ppm or 0.00005 %, an order of a magnitude lower than that reported by the FAO [27]. In our study, *B. bovis* lice from 3 farms tolerated exposure to 1 % deltamethrin and *B. bovis* lice from all 4 farms tolerated exposure to 0.5 % deltamethrin, both of which are significantly greater concentrations than those specified by the FAO or Boray and colleagues. Using a commercial 1 % deltamethrin pour-on product to assess *B. bovis* tolerance *in vitro*, Sands and colleagues reported high levels of louse mortality in response to the undiluted product [20]. However, the authors attributed the high mortality rate to the suffocation of the lice by the caprylic triglyceride excipient rather than the susceptibility of the lice to deltamethrin. As we used laboratory-grade deltamethrin without oily excipients the louse mortality rates we observed were unequivocally due to the drug.

Because the dispersal of the insecticide through the coat of the treated animal is non-uniform [17, 28, 29], it has been suggested that the concentrations of deltamethrin that *B. bovis* are exposed to in *in vitro* bioassays are probably considerably higher than the concentrations that the lice are exposed to *in vivo* [20] and that *in vitro* testing is more reliable than *in vivo* testing [17]. We found that observations during repeat visits of one farm (farm 1) before and after treatment with a deltamethrin-based pour-on correlated well with the bioassay results.

*B. equi* lice proved to be an excellent control for the bioassay as they were found to be fully susceptible to deltamethrin. Similar results were reported in a French *in vivo* study in a group of horses infested with *B. equi* [30]. There are currently no commercial deltamethrin-based louse management products licensed for use in horses in Ireland [31] which probably accounts for the high susceptibility of *B. equi* lice to this ectoparasiticide.

Chewing lice are probably more prone to developing resistance than sucking lice because they are less affected by systemic treatments. Insecticides that are applied to the animal’s coat disperse non-uniformly, probably resulting in the exposure of lice to sub-lethal concentrations and facilitating the emergence of pyrethroid-tolerant louse populations [17]. Moreover, the ability of *Bovicola* species to reproduce by parthenogenesis is also considered to be a major contributor to the emergence of insecticide resistance in *Bovicola* lice [17].

The widespread (over)use of pyrethroid-based products has led to the emergence of deltamethrin-resistant *B. bovis* lice in the UK [20], cypermethrin, alphacypermethrin and deltamethrin- resistant *B. ovis* lice in Australia [16, 32, 33] and cypermethrin and permethrin resistant *B. ocellatus* lice in the UK [17].

In comparison to the risks and the management of resistant populations of endoparasites such as parasitic nematodes and flukes, pesticide resistance in ectoparasites has not been given the same level of attention. Current methods for insecticide susceptibility testing are also less practical and limited in comparison to endoparasites, where laboratory tests such as the faecal egg count reduction tests are easily accessible, providing farmers with accurate information on the occurrence of endoparasite resistance on their farm. Moreover, providing advice on how to avoid resistance development within these populations remains a significant challenge, particularly with regard to obligate parasites such as lice. Firstly, when implementing integrated pest management strategies, the official advice is to use non-chemical treatment alternatives where possible [26]. However, there are very few non-chemical alternatives available to manage infestations of lice in comparison to other ectoparasites such as ticks, who can be targeted within the environment by means of pasture and grazing management. Secondly, current advice on how to manage resistant louse populations is quite conflicting and confusing. For example, official guidelines state that in order to reduce the incidence of insecticide resistance within louse populations, farmers should avoid an annual ‘blanket treatment’ of the entire herd in order to avoid unnecessarily treating un-infested animals [26]. However, in the same document it is also noted that by only treating the animals that are presenting with clinical signs of lice infestation, an effective reduction of overall louse populations within the herd is unlikely, as lice from untreated animals will spread to treated animals, re-establishing the infestation and necessitating subsequent treatments. Moreover, rotation between chemical classes as recommended to avoid resistance development is difficult due to the limited number of chemical classes that are licensed to treat cattle lice both in Ireland and abroad. Therefore, implementing this strategy is difficult and its effectiveness is limited, particularly when resistance to one chemical class has already occurred.

It is clear that in order to reduce the reliance of farmers on chemical treatments for treating infestations of lice, non-chemical alternatives should be made readily available. Future studies are warranted in order to evaluate the viability and functionality of non-chemical treatments such as essential oils for treating infestations of lice in cattle. Moreover, straight-forward and easy-to-follow advice on sustainable control measures for lice and avoidance of insecticide tolerance needs to be developed. Routine ectoparasiticide susceptibility testing, similar to what is available for endoparasites of livestock should also be made readily accessible to farmers. This will not only allow for a better understand of the levels of ectoparasite insecticide resistance occurring on the farm but could also serve to improve productivity and animal welfare.

In order to slow the emergence of insecticide-resistant populations of lice amongst cattle herds, the management of cattle during the winter housing period should be considered holistically. According to official guidelines, overcrowding of animals within sheds should be avoided where possible during the winter housing period, as the crowding of animals within sheds can play a role in the increase in louse numbers during this period [26]. Whilst we recognize that this may not be a feasible louse control strategy for some producers, we feel that it is important to note that avoiding overcrowding during the winter housing period is one of the few non-chemical louse control strategies currently available. Anecdotal evidence collected from the participants in our study suggested that the use of hair clipping along the topline of the animal may also serve as a non-chemical method of louse control however at present there is no scientific evidence available to confirm these findings.

It is recommended that all newly purchased stock are quarantined and treated for lice upon arrival to the farm. When treating animals for lice, care should be taken to ensure that dosing is carried out correctly by following the manufacturers’ guidelines. With regards to the use of Macrocyclic Lactone-based products, the product user should ensure that each animal is treated according to their weight and should regularly check that the dosing device is calibrated correctly to prevent underdosing [26].

 It is important to note that the results of the study are somewhat biased due to the opportunistic sampling method, the relatively small sample size and the deviations in the number of animals sampled on each participating farm. Therefore, future studies including a larger, more representative number of farms are warranted in order to gain a better understanding of the prevalence of lice species and infestations in cattle (including dairy) and the levels of ectoparasiticide tolerance in Irish cattle lice. Despite this, this study presents new data on the emergence of insecticide-resistance populations of lice and to our knowledge, the first record of deltamethrin resistance in *B. bovis* lice in Irish cattle.

## Conclusions

The occurrence of pediculosis in cattle herds is associated with significant production losses and animal welfare concerns. With the emergence of insecticide-tolerant populations of lice, information on the prevalence and control of louse infestations is important in order to ensure that infestations can be appropriately controlled and the spread of insecticide resistance slowed.

Our study provides new information on the cattle louse species present in Ireland and the control measures used by Irish farmers. Furthermore, we provide evidence that pyrethroid-tolerant populations of *B. bovis* lice are also emerging in Ireland, adding to existing reports of insecticide tolerance occurring globally in livestock lice.

## Data Availability

Not applicable.

## Abbreviation

FAO: The Food and Agriculture Organization of the United Nations
